# *In Vivo* Detection of Human TRPV6-Rich Tumors with Anti-Cancer Peptides Derived from Soricidin

**DOI:** 10.1371/journal.pone.0058866

**Published:** 2013-03-15

**Authors:** Chris V. Bowen, Drew DeBay, H. Stephen Ewart, Pamela Gallant, Sean Gormley, T. Toney Ilenchuk, Umar Iqbal, Tyler Lutes, Marzia Martina, Geoffrey Mealing, Nadine Merkley, Sandra Sperker, Maria J. Moreno, Christopher Rice, Raymond T. Syvitski, John M. Stewart

**Affiliations:** 1 National Research Council of Canada, Institute for Biodiagnostics (Atlantic) - Neuroimaging Research Laboratory, Halifax, Nova Scotia, Canada; 2 National Research Council of Canada, Institute for Marine Biosciences, Halifax, Nova Scotia, Canada; 3 Soricimed Biopharma Inc., Sackville, New Brunswick, Canada; 4 National Research Council of Canada, Institute of Biological Sciences - Neurobiology Program, Ottawa, Ontario, Canada; The Chinese University of Hong Kong, Hong Kong

## Abstract

Soricidin is a 54-amino acid peptide found in the paralytic venom of the northern short-tailed shrew (*Blarina brevicauda*) and has been found to inhibit the transient receptor potential of vallinoid type 6 (TRPV6) calcium channels. We report that two shorter peptides, SOR-C13 and SOR-C27, derived from the C-terminus of soricidin, are high-affinity antagonists of human TRPV6 channels that are up-regulated in a number of cancers. Herein, we report molecular imaging methods that demonstrate the *in vivo* diagnostic potential of SOR-C13 and SOR-C27 to target tumor sites in mice bearing ovarian or prostate tumors. Our results suggest that these novel peptides may provide an avenue to deliver diagnostic and therapeutic reagents directly to TRPV6-rich tumors and, as such, have potential applications for a range of carcinomas including ovarian, breast, thyroid, prostate and colon, as well as certain leukemia's and lymphomas.

## Introduction

In North America, 1 in 70 women will develop ovarian cancer within their lifetime. Despite recent advances with identification, ovarian cancer still remains difficult to detect and is typically identified only after it has metastasized to other parts of the body [1]. Prognosis for early stage detection is between 70% to 100% survival rates; however, 70% of cases are diagnosed at advanced stages where the 5-year survival rate is only between 15% and 25% [2]. Currently, no reliable non-invasive detection test exists for ovarian cancer.

Calcium concentrations are carefully regulated within cellular compartments through the integrated action of various membrane channels and pumps. Over the last several years certain members of a family of calcium ion influx channels, termed TRPV channels, have emerged as potential cancer biomarkers that may prove valuable for the development of improved tumor detection and/or targeted drug therapy. In 1999 novel calcium channels were reported in rabbit kidney tubules [3] and rat digestive tract [4] termed ECaC1 and CaT1, respectively, which resembled the capsaicin receptor (vanilloid receptor, VR1) [5]. ECaC1, CaT1, and VR1 turned out to be related to the transient receptor potential (TRP) calcium channel in *Drosophila* that mediates photoreception [6]–[8]. To date at least 30 TRP channel homologs have been identified in mammals, which are divided into six main subfamilies: TRPA (ankyrin), TRPC (canonical), TRPM (melastatin), TRPML (mucolipin), TRPP (polycystin) and TRPV (vanilloid) [9]–[11]. The TRPV sub-family consists of six members named TRPV1 to TRPV6. The first four channels are closely related and have roles in sensing various inputs including stretch, heat, acidity, noxious stimuli (nociception) and pain [9], [11]. These channels exhibit relatively low selectivities for calcium ion (P_Ca_/P_Na_ ∼1 to ∼15). In contrast, TRPV5 and TRPV6 are highly calcium ion-selective (P_Ca_/P_Na_∼100). Both of these channels are expressed in apical membranes of various tissues including kidney, intestine, pancreas and prostate [12]–[14]. For example, TRPV5 is expressed in the distal tubules of kidney where it functions in the reabsorption of calcium ion from pre-urine [12]; TRPV6 is predominant in the gastrointestinal tract where it is involved in apical entry of calcium [14].

TRPV6 is implicated in tumor development and progression [15]–[18]. The TRPV6 protein is over-expressed in carcinomas of ovary and other cancer such as breast, colon, prostate and thyroid [14]. TRPV6 mRNA is elevated in various tumor cell lines including those of colon, human leukemia and prostate [19]–[23]. In prostate cancer, TRPV6 mRNA levels are positively correlated to tumor progression and aggressiveness as indicated by Gleason score, pathological stage and extra-prostatic metastases [20], [24]. Indeed, TRPV6-positive prostate tumors have poor prognosis due to a propensity to invade surrounding tissues [25].

The link between tumor growth and over-expression of TRPV6 may involve the potentiation of calcium-dependent cell proliferation and inhibition of apoptosis. Schwarz et al. [26] showed that low doses of econazole, a capacitive calcium inflow blocker, reduced both calcium inflow signals and cell proliferation in HEK-293 cells transfected with TRPV6. In the LNCaP prostate cancer cell line, up-regulation of TRPV6 augments proliferation and cell survival while retarding apoptosis through a mechanism that appears to involve activation of the nuclear factor of activated T-cell (NFAT) transcription factor [27]; reduction of TRPV6 with siRNA reduced proliferation and increased apoptosis. In breast cancer cells, tamoxifen reduces expression of TRPV6 and calcium signaling, an effect that may partly explain the anti-cancer effects of this estrogen antagonist [28]. Moreover, knockdown of TRPV6 with siRNA improved the effectiveness of tamoxifen. Thus as suggested in a recent publication, inhibition of the TRPV6 channel offers a novel therapeutic strategy for the treatment of tumors over-expressing the TRPV6 channel [29].

Soricidin (accession number P0C2P6) is a novel paralytic peptide isolated from the submaxillary saliva glands of the northern short-tailed shrew (*Blarina brevicauda*; one of the most common mammals in Atlantic Canada) and, reported to inhibit calcium uptake *via* TRPV6 channels [30]. Subsequently, two peptide sequences from the C-terminus of soricidin (SOR-C13 and SOR-C27; Table 1) were shown to bind TRPV6 in ovarian cancer cells with high affinity [31]. Herein, we described the TRPV6 binding properties of SOR-C13 and SOR-C27 and the ability of these peptides to target human ovarian tumors in a xenograft mouse model. By conjugating the peptides with a fluorescent dye or a magnetic resonance imaging (MRI) contrast agent, we were able to monitor the bio-accumulation and bio-distribution of the peptides *in vivo*, ultimately imaging tumors expressing TRPV6. These findings open new diagnostic and/or therapeutic avenues for the early detection and treatment of ovarian tumors and potentially other TRPV6-rich tumors including those of breast, colon, prostate and thyroid, as well as certain leukemia's and lymphomas.

**Table 1:** Amino acid sequences of parent soricidin (accession number P0C2P6), SOR-C27 and SOR-C13.

Peptide	Sequence	Activity	Calculated Molecular Mass g mol^−1^
Soricidin	DCSQD CAACS ILARP AELNT ETCIL ECEGK LSSND TEGGL CKEFL HPSKV DLPR	Pain/ Cancer	5812.66
SOR-C27	EGK LSSND TEGGL CKEFL HPSKV DLPR	Cancer	2957.33
SOR-C13	KEFL HPSKV DLPR	Cancer	1568.85

## Materials and Methods

### The peptides

The sequences of soricidin, SOR-C13 and SOR-C27 peptides are shown in Table 1. The peptides were prepared by CanPeptide (Montreal, Canada) by solid phase synthesis. SOR-C13 and SOR-C27 showed the expected molecular mass by time-of-flight mass spectrometry (M^+^H^+^ of 1566.42 and 2958.02) with peptide purity (by HPLC) of 96.9% and 96.8% respectively. The peptide content of the lyophilized white powder (trifluoroacetate salt) was 83.5% and 65.2%. Subsequent peptide concentrations were calculated based on the free-base peptide.

### Solution NMR structure of SOR-C27

#### Sample Preparation

Nuclear magnetic resonance (NMR) spectroscopic data was acquired to determine the conformational structural features of the SOR-C27 peptide under various conditions. A stock solution (sample #1) of 10 mM SOR-C27 was prepared in 90/10 H_2_O/D_2_O with 50 mM potassium phosphate buffer at pH 6.6. From sample #1, three additional samples were prepared: #2) stock solution with 200 mM NaCl, #3) stock solution with 100 mM dodecylphosphocholine (DPC) and #4) stock solution diluted to 2 mM with 100 mM sodium dodecylsulfate (SDS). Sample #1 was acquired at temperatures ranging from 274 to 308 K. The peptide was completely soluble in all solutions and under all conditions.

#### NMR Spectroscopy

For samples 1–3 ^1^H NMR data sets were collected on a DRX-500 spectrometer (Bruker BioSpin AG, Fällanden, Switzerland) operating at 500.13 MHz using a 1 mm OD H[CN] probe. For sample 4, ^1^H (and indirectly ^13^C and ^15^N) NMR data sets were collected on an AVANCEIII 700 spectrometer operating at 700.13 MHz using a 1.71 mm OD HC[N] probe. ^1^H chemical shifts were referenced to 2,2-dimethyl-2-silapentane-5-sulfonate through the water resonance calibrated at each temperature. ^13^C and ^15^N chemical shifts were indirectly referenced through the ^1^H referencing.

For structural determination with samples 1 and 4, phase-sensitive 2D ^1^H-^1^H NOESY data sets (120, 250 and 400 ms mixing time) and ^1^H-^1^H TOCSY (MLEV17; 120 ms mixing time, sample 4 DIPSI-2; 90 ms mixing time) data sets were recorded with a pre-saturation or a soft pulse for water suppression. Data sets were collected with 1024×380 complex points, apodized with a 70° shifted sine-squared-bell window function in both dimensions, zero filled and linear predicted in the indirect dimension to give a final 2D spectrum of 2048×2048 real points.

#### Structural Calculations

NMR spectra were analyzed, and peak positions and volumes determined using Sparky 3.110 software operating on a Linux PC system. For structural determination, all spin systems were identified through chemical shifts and characteristic TOCSY cross peak patterns. Side chain and back bone resonances of Asn7, Thr9, Phe17 and Val23 were readily assigned from their unique TOCSY patterns and each residue only occurs once in the sequence. Starting from these residues, sequence-specific assignments of backbone and side chain resonances were determined using standard methods, *i.e.* starting from the readily assigned residues, adjacent residues were sequentially determined by following the H^α^ *_i_*-H^N^ *_i+1_* and H^N^ *_i_*-H^N^ *_i+1_* NOESY connections. Once adjacent residues were identified, longer-distance NOESY connections were assigned. For structure calculations of SOR-C27 in buffered water, DPC and SDS micelles, distance restraints were determined from integration of the cross-peaks from the 250 ms (sample 4; 200 ms) NOESY spectrum, and classified into four groups: strong, medium, weak and very weak corresponding to inter-proton distance ranges of <2.3, 2.0–3.5, 3.3–5.0, and 4.8–6.0 Å, respectively.

All structural calculations were based on previous studies using the XPLOR 3.1 software package. Briefly, an initial random coil extended structure was used to generate a total of 50 embedded structures. Close contacts were removed by energy minimization of 1000 conjugate gradient steps before proceeding to the restrained simulated annealing molecular dynamics (MD) calculations. Eight steps comprising a total simulation time of 120 ps were used for the MD simulations. Initially, the system was set to 1500 K and all force constants for bonded, NOESY and non-bonded interactions were scaled to 10% of their full values. After the third step, the force constants had been linearly scaled to their full values. In the last five steps the temperature was decreased uniformly to 300 K. Calculated structures were then energy minimized with 2000 conjugate gradient steps.

Diffusion ordered spectroscopy (DOSY) is capable of precisely determining the diffusion constants of molecules in solution. DOSY data was acquired for the SOR-C27/SDS sample. The 3 kDa SOR-C27 peptides and the 80 kDa SDS micelles have similar diffusion rates consistent with strong binding of the peptide to the SDS micelles.

### Culture of cell lines

#### Transfection and overexpression of TRPV6 in HEK-293 cells

Human HEK-293 cells (6–9 passages) were plated in a 24-well plate at a density of 5×10^5^ cells per well, 24 hours before transfection. Each well was diluted in serum-free medium and incubated for 5 minutes at room temperature then combined slowly and incubated at room temperature. After a 20 minute incubation, cells were exposed to the transfection complex consisting of 0.5 µg of pdi-CaT-L plasmid DNA (a gift from Dr. V. Flockerzi, Experimentelle und Klinische Pharmakologie und Toxikologie der Universität des Saarlandes [23]) and 1.5 µl lipofectamine 2000 reagent. pdi-CaT-L is a bicistronic expression vector containing cDNA for the protein-coding region of TRPV6 and the mutated Green Fluorescent Protein (S65T; EGPF) separated by an internal ribosome expression site (β-actin promoter /KOZAK/TRPV6/IRES/EGFP in pCAGGS) [23]. The IRES was derived from encephalomyocarditis virus [32]. Transfection of HEK-293 cells with the bicistronic vector allows for simultaneous production of TRPV6 and EGFP and selection of fluorescent cells producing TRPV6 for the electrophysiology measurements. From previous investigations using this plasmid and other similar plasmids, it was determined that the TRPV6 channel is highly expressed, localized at the membrane surface, glycosylated and constitutively active giving rise to a Ca^2+^-invoked inward current [33]–[35].

#### Culture of cancer cell lines

All cell lines were obtained from the American Type Cell Collection and grown at the recommended conditions of 37°C in an atmosphere of 5% CO_2_. SKOV-3 (ATCC, HTB-77) is a tumor cell line originally derived from the ascetic fluid of a female subject with an ovarian tumor. They were cultured in McCoy's 5a medium containing 10% fetal bovine serum (FBS). DU145 (ATCC, HTB-81) is a tumor cell line derived from a brain lesion of a male subject with metastatic prostate cancer. They were cultured in Eagle's Minimum Essential Medium (ATCC) supplemented with 10% heat-inactivated FBS. SKOV-3 and DU145 are tumorigenic in CD-1 nude mice, forming primary ovarian and prostatic adenocarcinomas, respectively.

#### TRPV6 electrophysiology

Whole-cell patch clamp recordings were performed on HEK-293 cells expressing TRPV6 and EGFP. Transfected cells were readily identified from non-transfected cells using a fluorescent Axiovert 135 microscope (Olympus). The recordings were performed using an Axopatch 1D amplifier controlled and monitored using pClamp 9 software (MDS Analytical Technologies, Sunnyvale, CA, USA). Data sets were digitized at 20 kHz and filtered at 2 kHz. Patch pipettes were pulled from borosilicate glass using a P-80C pipette puller (Sutter Instrument, San Rafael, CA, USA), and had a resistances of 2–4 MΩ when filled with the pipette solution containing: 145 mM cesium-methane sulfonate, 8 mM NaCl, 1 mM MgCl_2_, 3.64 mM CaCl_2_, 10 mM HEPES and 10 mM EGTA (to buffer the intracellular Ca^2+^) with pH was adjusted to 7.2 using CsOH. The extracellular solution contained: 145 mM NaCl, 10 mM CsCl, 10 mM CaCl_2_, 2 mM MgCl_2_, 2.8 mM KCl, 10 mM HEPES and 10 mM glucose with pH adjusted to 7.2 using NaOH.

To isolate TRPV6 currents, HEK-293 and transfected HEK-293 cells were voltage-clamped at +50 mV with a 50 ms linear voltage ramp (−110 mV to +90 mV) protocol applied in the presence or absence of a TRPV6 inhibitor, La^3+^ ions (10 µM), SOR-C27, SOR-C13 or SOR-C27-Cy5.5. Although the La^3+^ ion is a non-specific inhibitor there is currently no known selective inhibitor of the TRPV6 channel. The ramp was averaged over 10 times at 2 s intervals between ramps.

Analysis was performed off-line using IGOR (WaveMetrics Inc., Lake Oswego, OR, USA) software. The statistical significance was determined with paired Students' t-test (two-tailed). All values are expressed as mean ± SEM with a *p*-value of <0.05 considered significant.

### Imaging studies

#### Production of tumors for imaging studies

Similar to previous investigations, CD-1 nude mice (6–8 weeks old, Charles River) were used for fluorescent labeling (male mice) and MRI (female mice) studies [36], [37]. Mice were housed in cages in groups of three to five, and maintained on a 12 hour light/dark schedule at 22°C and relative humidity of 50±5%. Sterilized food and water were freely available. Subcutaneous tumor implantation was performed in mice under light isofluorane anesthesia. SKOV-3 or DU145 (6×10^6^ cells/50 µl Matrigel dilution 1∶1 in saline) were implanted in the left flank of the mice used in the fluorescence study while 1×10^7^ SKOV-3 cells were injected in mice used in the MRI study. The size of the tumors, calculated using the formula length × width^2^/2, was monitored with calipers. Over a 6 week period, tumors were grown to a maximum size of 300 mm^3^. This investigation was carried out in strict accordance and compliance with Canadian Council on Animal Care. The protocol was approved by the Animal Care Committee of Dalhousie University (protocol #09-047). All surgery was performed under appropriate anesthesia and all efforts were made to minimize suffering.

#### Preparation of SOR-C27-Cy5.5 for Fluorescent Studies

SOR-C27 (6.64×10^−4^ mmole) was derivatized at the sole Cys14 thiol group by reacting with a 25% excess of the maleimide derivative of Cy5.5 (8.86×10^−4^ mmole; GE Healthcare Amersham) according to the provided instructions. The tagged peptide was separated from unreacted components using a Sephadex G-25 size exclusion column (20×1.5 cm). Fractions containing the tagged peptide were lyophilized and purity confirmed with HPLC.

Since SOR-C13 does not contain a single unique conjugation site, coupling the peptide to a Cy5.5-NBS ester (GE Healthcare Life Sciences. Baie d'Urfe, QC) resulted in a mixture of tagged peptides through Lys1 and Lys8. Consequently the binding and fluorescents results could be skewed and thus, only preliminary *in vivo* investigations were performed.

#### Preparation and characterization of SPIO-(SOR-C27)_x_ for MRI studies

Monitoring biodistribution by MRI relies on a contrast agent such as super-paramagnetic iron oxide (SPIO) beads to be chemically attached to the compound of interest SOR-C27 (SOR-C13 was not utilized as it did not contain a single unique conjugation site). SPIO beads functionalized with maleimide groups (MicroMod 77-96-201) were used without further purification. Beads were reacted with a 5-fold molar excess of freshly prepared and buffered SOR-C27 (1 mM, 50 mM PBS, pH 7.2) for 1 hour at room temperature. The mixture was diluted with 50% methanol and centrifuged at 400 rcf until the supernatant had a slight red colour affording a pellet of SOR-C27 functionalized SPIO beads. The SPIO-peptide pellet was washed and re-suspended in sterile Dulbecco's PBS (DPBS) to a concentration of 2.5 mg Fe/ml for injection into mice. For the control, SPIO beads were prepared by reaction of the maleimide-functionalized beads with a freshly prepared cysteine solution using an equivalent protocol. The cysteine blocks maleimide groups on the beads rendering them non-reactive.

The number of SOR-C27 peptides per bead was determined by quantitative ^1^H NMR analysis of the supernatant to determine number of un-reacted peptide molecules. On average 75 SOR-C27 molecules were conjugated to each SPIO particle. Conjugation of the peptide to the beads was verified by trypsin digestion of the bead suspension and analysis the liberated peptide fragments by mass spectrometry. After digestion and removal of the beads by centrifugation, the resultant supernatant contained two peptide fragments with sequences consistent with cleavage of the SOR-C27 peptide.

#### Fluorescent imaging set-up and data collection

All optical imaging experiments were performed using a small-animal time-domain eXplore Optix MX2 pre-clinical imager, and images were analyzed or reconstructed as fluorescence concentration maps using ART Optix Optiview analysis software 2.0 (Advanced Research Technologies, Montreal, QC). A 670-nm pulsed laser diode at a repetition frequency of 80 MHz and a time resolution of 12 ps light pulse was used for excitation. The fluorescence emission at 700 nm was collected by a highly sensitive time-correlated single photon counting system and detected through a fast photomultiplier tube. The data were recorded as temporal point-spread functions (TPSF). For imaging, isofluorane anesthetized mice were positioned on an animal stage within a chamber that allowed for maintenance of gaseous anesthesia.

After tumors reached a volume of ∼300 mm^3^, SOR-C27-Cy5.5 (100 µg) was injected intraperitoneal (i.p.) into TRPV6-positive (SKOV-3) xenograft tumor bearing mice, and optically imaged at multiple time intervals (0.5, 1, 2, 4 and 24 hours) post injection. For competition studies, untagged SOR-C27 peptide (10 mg) was injected 10 minutes prior to the injection of Cy5.5 tagged SOR-C27 (100 µg). At the end of the imaging, animals were sacrificed under deep anesthesia by intracardiac perfusion using saline. The organs and tumor were excised and scanned *ex vivo* with the optical imager.

#### Fluorescent imaging processing and analysis

ART Optix Optiview analysis software was used to estimate the fluorescence decay rate. The software de-convoluted the measured fluorescent intensity-time decay curve using the Levenberg-Marquardt algorithm, which applies a nonlinear least-squares minimization algorithm to compute the coefficients of a multi-exponential expansion of the fluorescence decay. A two-exponent fitting was used, and long (τ_1_) and short (τ_2_) fluorescence lifetime components, together with their weighted average value (τ_av_), were automatically calculated according to the following equation:
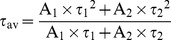



where A_1_ represents the amplitude of the first exponential fluorescence and A_2_ represents the amplitude of the second exponential fluorescence.

#### Magnetic resonance imaging methodology

All MRI scans were performed on a Magnex Scientific 3 Tesla clinical head MR scanner (Oxford, UK) retrofitted for small animal imaging, using a Magnex Scientific gradient coil (inner diameter of 21 cm; maximum operating gradient strength of 200 mT/m) interfaced with a Varian Inc. Direct Drive spectrometer (Palo Alto, CA). A 25 mm ID ‘Litzcage’ quadrature RF coil (Doty Scientific, Columbia, SC), tuned to 128.8 MHz, was used as a transmit/receive volume coil for imaging.

*In vivo* images were obtained using a 3D true-FISP (T2/T1 weighted) imaging sequence. Repetition time (T_R_), echo time (T_E_), flip angle and bandwidth (BW) were optimized for best image quality. The sequence consisted of T_R_/T_E_ = 8/4 ms, flip angle  = 30° and BW = 50 kHz. A field of view (FOV) of 38.4×25.5×25.5 with matrix dimensions 256×170×170 was used to acquire 150-µm^3^ isotropic spatial resolution images with 6 signal averages (∼48 minutes per MRI scan). This FOV allowed for simultaneous imaging of tumor site as well as inguinal and popliteal lymph nodes.

Mice were anaesthetized with a loading dose of 3% isofluorane (in 100% oxygen) in an induction chamber, and were transferred to an integrated nosecone and RF coil imaging sled (developed in house) to be maintained at 1.5–2% isofluorane for the duration of the MRI scan. The respiratory rate and internal body temperature of the mice were monitored using an MRI compatible physiological monitoring and gating system (SA Instruments Inc, Stony Brook, NY). The internal body temperature of the mouse (measured rectally) was maintained at 37±1°C via temperature control feedback loop and air heating system.

Mice were injected with ∼300 µL corresponding to approximately 24 mg Fe/kg body weight of either cysteine blocked SPIO beads (CYS-SPIO; control, *n* = 9) or SOR-C27 conjugated beads (SOR-C27-SPIO; *n* = 9) by either intravenous (i.v.) or i.p. injection and imaged at 2 hours (day 0) and 26 hours (day 1) post-injection. Baseline scans (day -1) of each mouse were also performed prior to injections to allow proper comparison of tumor and lymph node morphology and contrast pre- and post-injection. After the MRI time-course, animals were sacrificed and tumors were immediately excised for histological/biochemical analysis.

#### MRI Image Processing

All images were first zero-filled using ImageJ (NIH) software. Images were co-registered in RView (Colin Studholme, University of California) for each mouse. A semi-automated volume segmentation routine was implemented via segmentation in RView to accurately determine tumor volumes.

## Results

### Solution NMR structure of SOR-C27

**Figure 1:**
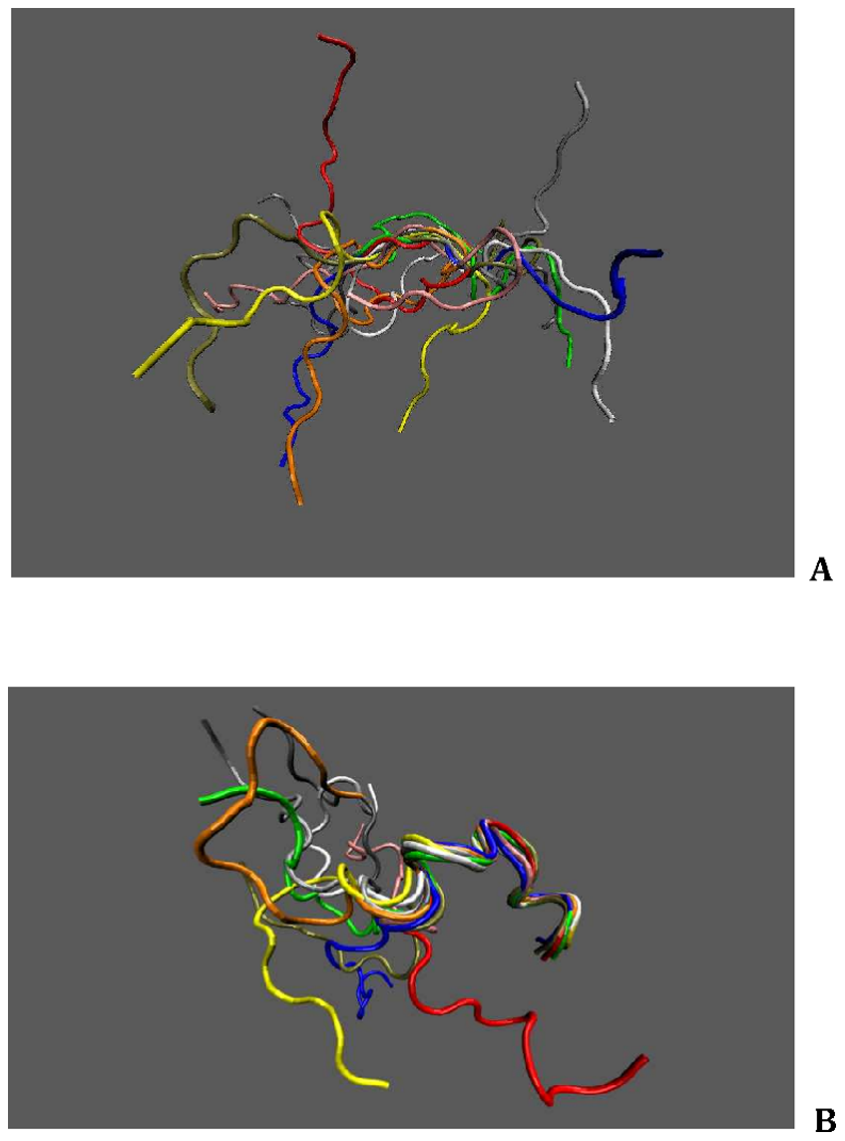
Lowest energy NMR solution structures of SOR-C27. A representative NMR solution structure of 10 mM SOR-C27 in 50 mM potassium phosphate buffer, 200 mM NaCl or 100 mM dodecylphosphocholine (**A**). A representative NMR solution structure of 2 mM SOR-C27 in anionic 100 mM sodium dodecylsulfate micelles showing induction of helical structure from residues 15 to 25 (**B**).

Information on SOR-C27 peptide secondary structure was derived by examining H_α_ chemical shifts under three different conditions, 10 mM phosphate buffered water, sodium dodecylsulphate (SDS) and dodecylphosphocholine (DPC). Structural calculations utilizing nuclear Overhauser effect (nOe) distance restraints for 10 mM SOR-C27 in buffered water, NaCl or DPC (Fig. 1A) generated a total of 40 lowest-energy structures each that had no violations of NOESY constraints >0.5 Å. Since no portion of the peptide could be identified as adopting a regular structure, structural validation checks were not performed. However, in SDS micelles changes in the amide ^1^H chemical shifts were observed for residues Lys15, Phe17, Leu18, His19, Ser21, Val23, and Arg27 indicative of a structural change in the anionic environment. Structure calculation of SOR-C27 in 100 mM SDS micelles by means of nOe distance restraints afforded two turns of an α-helix (residues 15–25) that was consistent in the top 10 lowest energy structures of the 50 calculated (Fig. 1B).

### Electrophysiology of TRPV6 and SOR-C27 and SOR-C13

#### Wild-type HEK-293 cells do not express TRPV6 channels

**Figure 2:**
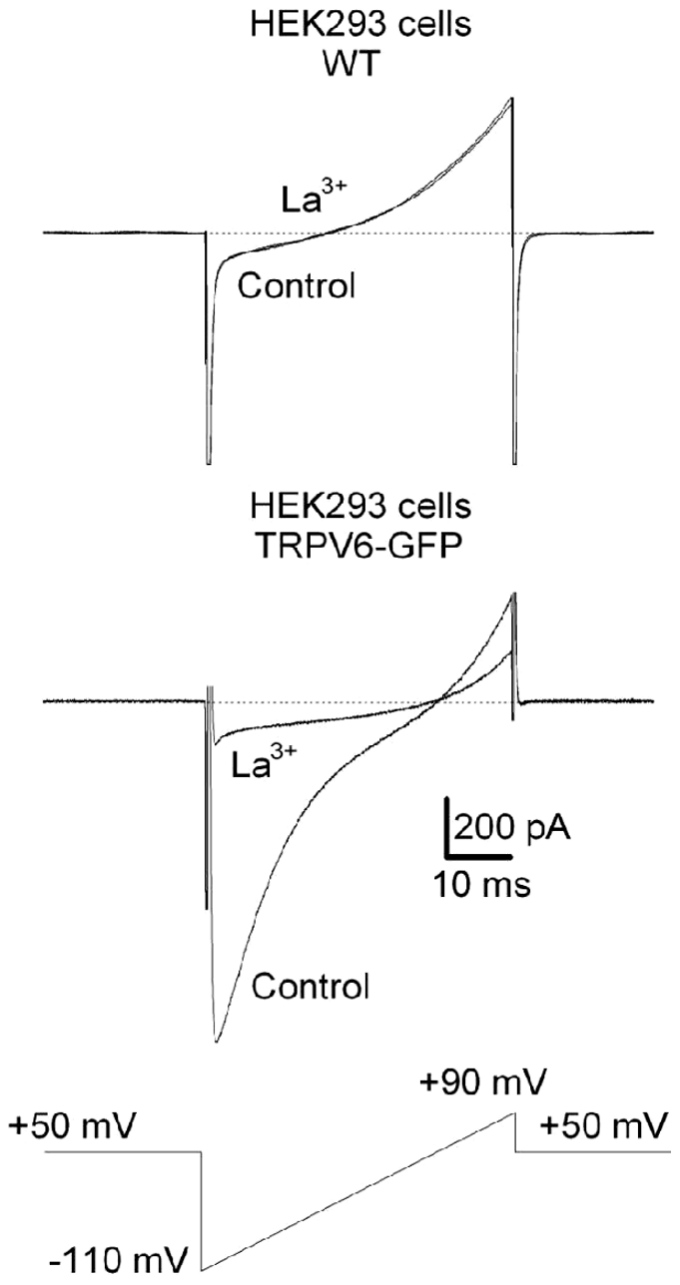
Comparison of electric currents from wild type HEK-293 cells and TRPV6/EGFP HEK-293 cells. The measured currents in wild type HEK-293 cells are inductive of no over-expression of TRPV6 channels. For TRPV6/EGFP HEK-293, the absence of La^3+^ ions showed a large current with the current being efficiently blocked in the presence of La^3+^ ions. Effect of La^3+^ ions (10 µM) on currents evoked by a 50 ms ramp (bottom) in wild type HEK-293 cells and TRPV6/EGFP HEK-293 cells.

Electrophysiology experiments were carried out in the HEK-293 cells transfected with TRPV6 channels. First, to rule out the presence of TRPV6 channels in wild type HEK-293 cells, whole-cell patch clamp recordings with a 50 ms linear voltage ramp (−110 mV to +90 mV) was applied in the absence or presence of La^3+^ ions (10 µM). Lanthanum ions, which inhibit TRPV6 current, had no effect on the current amplitudes evoked by the ramp (control: 106±27 pA; La^3+^ ions: 111±13 pA, *n* = 3; *p*>0.05; Fig. 2). Whole-cell patch clamp recordings of HEK-293 cells transfected with TRPV6/EGFP were acquired in the absence or presence of La^3+^ ions (10 µM). Lanthanum ions reduced the amplitudes of the TRPV6 currents evoked by the ramps by 82±5% (control: 1065±450 pA; La^3+^ ions: 224±105 pA; *n* = 3; Fig. 2). The amplitudes of the currents evoked by the ramp in wild type HEK-293 (106±27 pA, *n* = 3) were significantly smaller (*p* = 0.002) than currents in TRPV6/EGFP transfected HEK-293 cells (1065±450 pA, *n* = 3).

#### SOR-C13 and SOR-C27 reduce the amplitude of the TRPV6 currents

Similar investigations were performed to characterize the effect of increasing concentrations of the peptides SOR-C13 and SOR-C27 on TRPV6 channels. In the presence of 83.5 nM, 417.5 nM, 835 nM and 25 µM SOR-C13 the amplitude of the TRPV6 currents was reduced by 18±4.0% (*p*<0.05, *n* = 9), 22±4% (*p*<0.05, *n* = 9), 24±4% (*p*<0.05, *n* = 7), and 25±5% (*p*<0.05, *n* = 7), respectively (Figs. S1-A and -B). The IC_50_ (K_d_) of SOR-C13 was 14±1.3 nM (Fig. S1-C). Amplitudes remained depressed during successive ramps even with washout of peptide. Terminating the voltage ramps during peptide washout allowed recovery of the TRPV6 currents. Since it was not possible to washout the peptide under voltage ramp stimulation, the K_d_ was estimated to be equal to the K_1_ (K_1_ = 14±1.3 nM).

**Figure 3:**
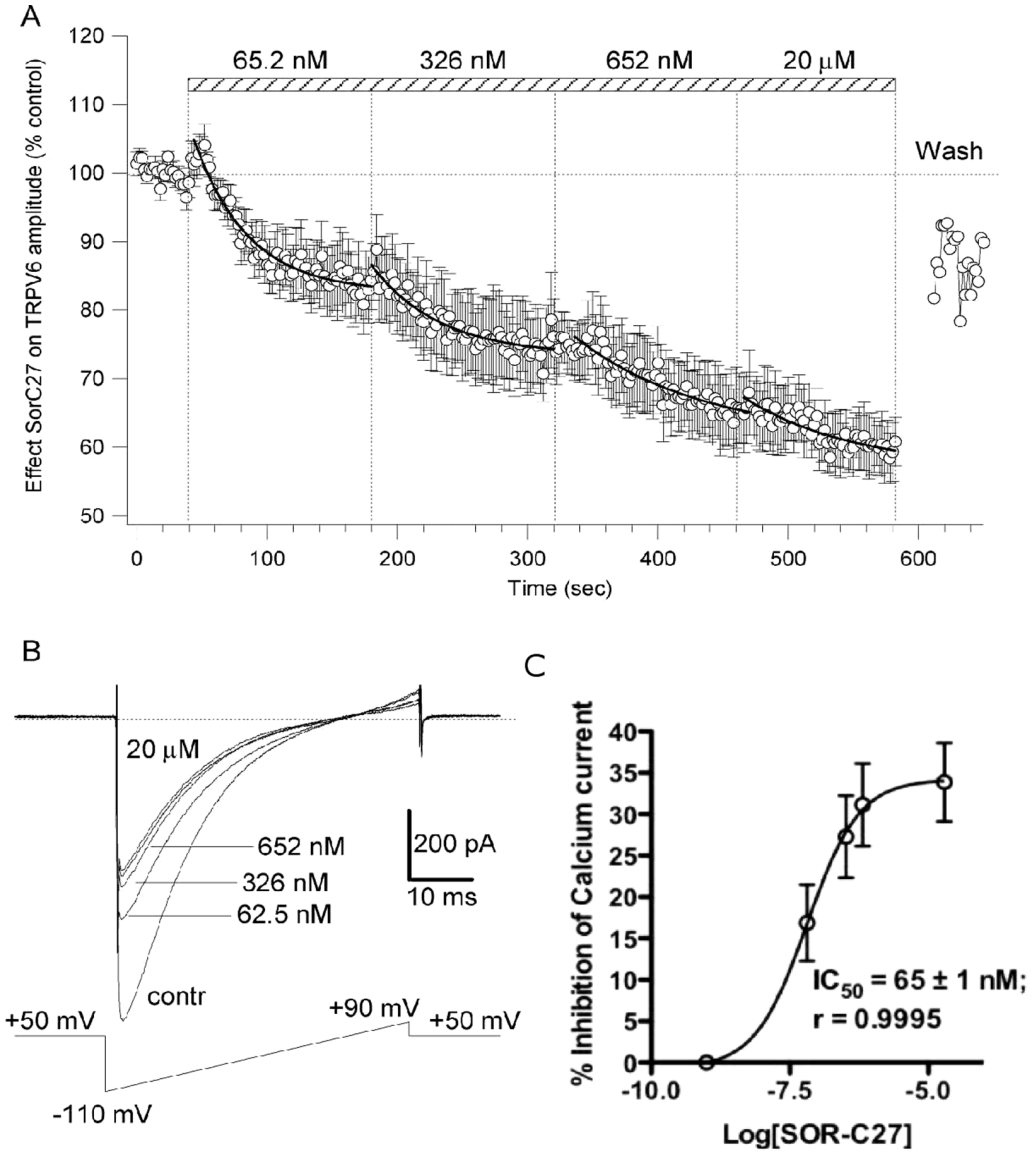
SOR-C27-mediated reduction of TRPV6 current amplitude in TRPV6/EGFP HEK-293 cells. The time course of the SOR-C27-induced reduction in TRPV6 current amplitude for normalized TRPV6 current amplitudes (%) are plotted as a function of time for SOR-C27 concentrations of 65.2 nM, 326 nM, 652 nM and 20 µM (*n* = 5) (**A**). The bar indicates the period of SOR-C27 application. Examples of traces of TRPV6 currents with each trace being an average of 10 traces (**B**). Dose–response curve for SOR-C27 with error bars representing SEM (**C**).

With successive exposures to 65.2 nM, 326 nM, 652 nM and 20 µM SOR-C27, the maximum amplitude of the TRPV6 currents was reduced by 17±4% (*p*<0.05), 27±5% (*p*<0.05), 31±5% (*p*<0.05), and 34±5% (*p*<0.05), respectively (*n* = 5; Figs. 3A and B). The SOR-C27 effect was mediated in a concentration-dependent manner with an IC_50_ (K_d_) value of 65±1 nM (Fig. 3C). Similar to the washout protocols used with SOR-C13, amplitudes remained depressed and recovery of the TRPV6 currents was only observed after terminating the voltage ramps. Again, the K_d_ was estimated to be equal to the K_1_ (K_1_ = 65±1 nM).

Conjugation of the fluorescent dye Cy5.5 with the SOR-C27 peptide modified the blocking properties of SOR-C27 to the TRPV6 channels. SOR-C27-Cy5.5 (500 nM) reduced the amplitude of the TRPV6 currents in TRPV6/EGFP transfected HEK-293 cells by 13±6% (control: 611±343 pA; SOR-C27-Cy5.5 (500 nM): 566±340 pA; *n* = 5; *p*<0.05). The degree of inhibition of TRPV6 channels was significantly smaller for SOR-C27-Cy5.5 compared to SOR-C27 at 326 nM: 27±5% (SOR-C27) *vs.* 13±6% (SOR-C27-Cy5.5). Inhibition of TRPV6 channels by SOR-C27-Cy5.5 was not reversible.

### Bio-distribution of Cy5.5 derivatives of SOR-C27

#### IR Fluorescence imaging of xenografted tumors

**Figure 4:**
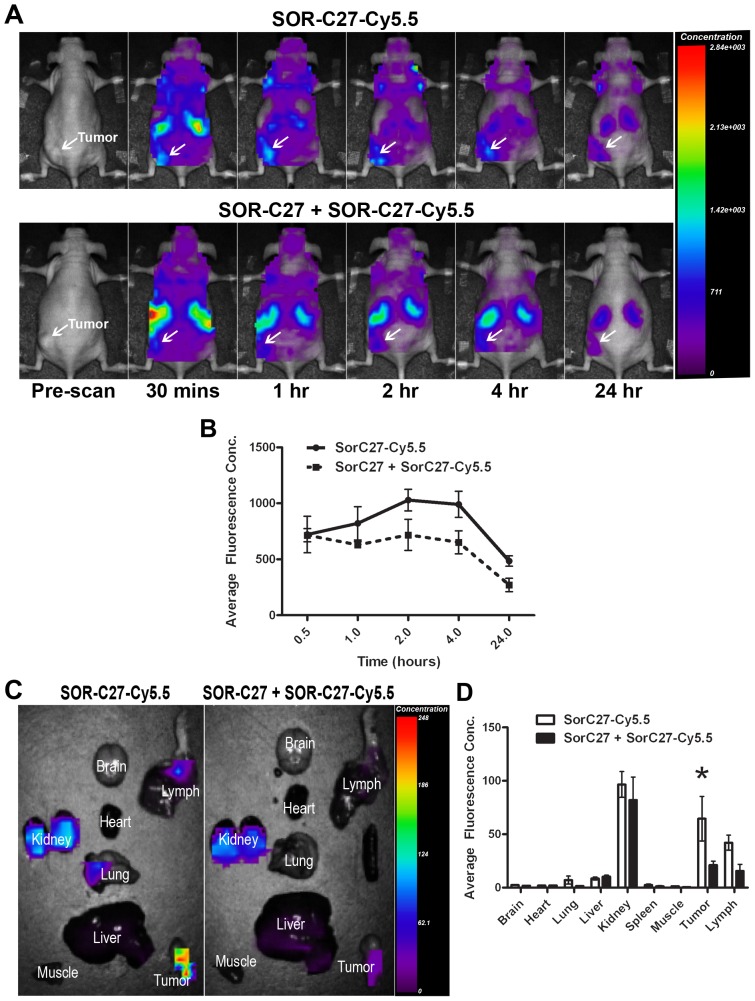
*In vivo* optical imaging of mice bearing SKOV-3 xenograft tumors after i.p. administration of 100 µg of SOR-C27 tagged with Cy5.5. Dorsal whole-body images at indicated time points after injection of either SOR-C27-Cy5.5 (upper panels of **A**) or SOR-C27-Cy5.5 competitively blocked by 100 fold excess of SOR-C27 (lower panels of **A**). Arrows indicate the location of the solid subcutaneous tumor in the flank of the animal. Graph illustrating the changes in average fluorescence concentration in the tumor at indicated times after injection of SOR-C27-Cy5.5 or SOR-C27-Cy5.5 competitively blocked by SOR-C27 (**B**). *Ex vivo* optical images of mouse organs 24 hours post-injection of SOR-C27-Cy5.5 alone (left panel of **C**) or SOR-C27-Cy5.5 competitively blocked by SOR-C27 (right panel of **C**). Graph illustrating the average fluorescence concentration imaged *ex vivo* in various tissues 24 hours post-injection of SOR-C27-Cy5.5 or SOR-C27-Cy5.5 competitively blocked by SOR-C27 (**D**). *Indicates significant difference between SOR-C27-Cy5.5 and SOR-C27-Cy5.5 competitively blocked SOR-C27-Cy5.5 (*p*<0.05). In **B** and **D**, data are expressed as mean ± SEM for *n* = 3 animals.

SOR-C27-Cy5.5 bio-distribution was evaluated after i.p. injection (100 µg) in mice bearing the ovarian (SKOV3) and prostate (DU145; Fig S1) tumor in the left flank using *in vivo* optical imaging. Fig. 4A (upper panel) shows representative *in vivo* images of whole-body scans of a mouse containing a SKOV-3 xenograft tumor at 0.5, 1, 2, 4 and 24 hour time points. Analysis of the average fluorescence concentration in the tumor region of interest (ROI) demonstrated that SOR-C27-Cy5.5 slowly accumulates in tumors over time with a peak of accumulation around 2 hours. The average fluorescence concentration was 721±325 A.U., 821±299 A.U., 1030±192 A.U., 991±234 A.U. and 484±93 A.U. at 0.5, 1, 2, 4 and 24 hours respectively (Fig. 4B). Low background signal (purple colour) was detected in the whole body due to the presence of circulating fluorescent dye that was eliminated over time. As expected, high intensity fluorescence signals were observed in kidneys, which is the main clearance route of the fluorescent tag.

For competition experiments, pre-injection of 100-fold excess of untagged SOR-C27 reduced the accumulation of SOR-C27-Cy5.5 peptide by approximately 30% 2–4 hours post-injection compared to SOR-C27 alone (Figs. 4A and B, lower panel; similar results with untagged SOR-C13, data not shown). The average fluorescence concentration for SOR-C27-cy5.5 in the competition experiment was 715±100.8 A.U., 628.7±51.43 A.U., 718.7±241.4 A.U., 651.7±176.9 A.U. and 270.3±104.3 A.U. for 30 mins, 1 hr, 2 hr, 4 hr and 24 hr, respectively (Fig. 4B). After 24 hours, animals were sacrificed and liver, kidney, spleen, lung, heart, brain, muscle, tumor and upper right arm lymph node area excised and imaged (Fig. 4C). The average fluorescence in the *ex vivo* tumors of animals injected with 100 µg SOR-C27-Cy5.5 alone was significantly higher (65±42 A.U.) than in those pre-injected with 10 mg untagged SOR-C27 followed by 100 µg SOR-C27-Cy5.5 (21±6 A.U.; Fig. 4D).

#### Fluorescence lifetime analysis

The *ex vivo* average fluorescence lifetime concentration and values from the SKOV-3 tumor, lymph nodes, kidney and liver are presented in Fig. S3. After fitting the data to a two-exponent model, both long (τ_1_) and short (τ_2_) fluorescence lifetime components, together with their weighted average value τ_av_, were obtained (Table 2). The τ_2_ represents the free Cy5.5 dye, whereas τ_1_ represents the Cy5.5 bound to the peptide. The weighted lifetime percentages indicated a trend for a higher percentage of the lifetime signal due to free Cy5.5 dye in elimination organs (61.5%, kidney; 58.3% liver) compared to a higher percentage of the lifetime signal due to Cy5.5 bound to peptide in the target organs (67.8%, tumor; 62.8%, lymph nodes). Digital Z-slices (2 mm) through mice bearing ovarian and prostate tumors at the level of the tumors are shown in Fig. S2. This manipulation provides a clear field of view of tumor fluorescence at the point of maximal tumor uptake of the tagged peptide (∼2 hours) apart from the fluorescent signal observed in kidneys.

**Table 2:** Long (τ_1_) and short (τ_2_) fluorescence lifetime components and their weighted average value τ_av_ (%) measured in *ex vivo* organs of mice bearing SKOV-3 xenograft tumors after injection with SOR-C27-Cy5.5 derived from a two-exponent model.

	Weighted Lifetime Average, τ_av_ (%)	
Tissue	Short lifetime (τ_2_)	Long Lifetime (τ_1_)
Tumor	1.15 ns (32.3%)	1.89 ns (67.8%)
Lymph Node	1.16 ns (37.2%)	1.96 ns (62.8%)
Kidney	0.58 ns (61.5%)	1.52 ns (38.5%)
Liver	0.32 ns (58.7%)	1.16 ns (41.3%)

### Magnetic Resonance Imaging of ovarian tumors

#### Visualization of ovarian tumors

As a second approach to study bio-distribution of SOR-C27 (SOR-C13 was not utilized as it did not contain a single unique conjugation site), we conjugated the peptide to SPIO beads that allowed monitoring by MRI. In these experiments involving mice bearing SKOV-3 tumors, clear imaging of tumor growth sites was achieved. Tumors displayed strong internal contrast indicative of cellular in-growth and tumor viability with well-defined outer margins, which allowed effective 3D segmentation of tumor sites (Fig. S4). Scan parameters yielded sufficient signal-to-noise ratio with values typically ranging from ∼30 to 40 across all animals in the study, which allowed key structures to be resolved and permitted accurate segmentation of tumor volumes.

#### SPIO and SPIO-(SOR-C27)_x_ Injections: Verification of Systemic Uptake

MRI allowed verification of the systemic uptake of SPIO within the lymphatic system up to 26 hours post-injection as evidenced by black signal voids observed within the left and right inguinal and popliteal lymph nodes (Figs. S5-A-C, S6 A–C). This feature was not present in lymph nodes in the pre-injection baseline scans (Figs. S5-A, S6-A). SPIO accumulation was observed in the lymph nodes 2 hours post-injection using both SOR-C27 functionalized SPIO beads (Fig. S6-B) and SPIO control beads (Fig. S5-B). Thus the appearance of SPIO within the lymphatic system occurs very rapidly, and is maintained for at least 26 hours, suggesting that both SPIO and SOR-C27-SPIO beads are readily recognized by the reticuloendothelial system.

#### SPIO visualization at tumor sites

**Figure 5:**
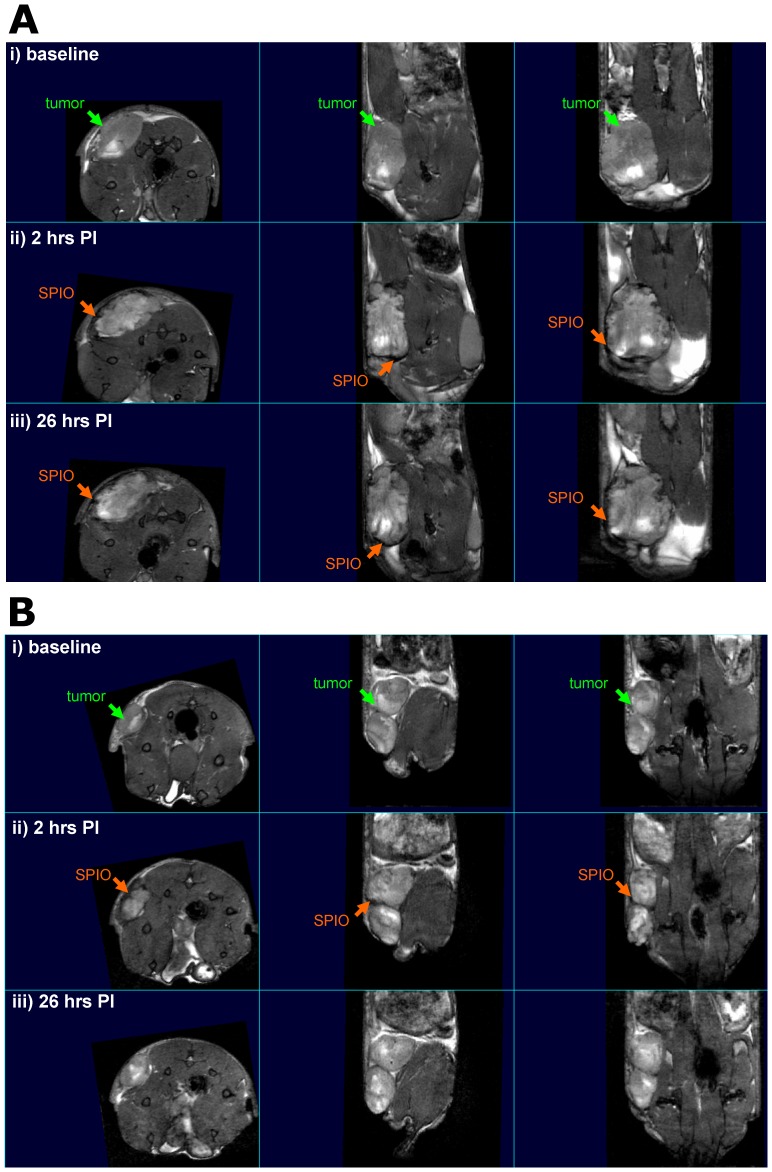
150 µm^3^ axial, saggital and transverse MRI images. A mouse injected with SOR-C27-SPIO at each MRI time point showing ***i*** baseline tumor site, ***ii*** the accumulation of SPIO at 2 hours post-injection and ***iii*** SPIO persistence at tumor site at 26 hours post-injection (**A**). A mouse injected with SPIO control bead at each MRI time point showing ***i*** baseline tumor site, ***ii*** the initial accumulation of SPIO at 2 hours post-injection and ***iii*** SPIO clearance at tumor site at 26 hours post-injection (**B**).

SPIO was apparent in tumors within 2 hours of animals receiving either control SPIO (n = 6) or SPIO-conjugated SOR-C27 (n = 3) peptide injections, and therefore, not a function of SOR-C27 at this post-injection time (Figs. 5A-*i,ii*, 5B-*i,ii*). SPIO presence manifested itself in the darkened appearance of the surface of the majority of tumors, although two tumors showed evidence of SPIO concentrated towards the tumor center. SPIO visualization soon after injection is not surprising, as one would expect to see long-circulating blood-pool SPIO contrast agents retained within the vasculature after injection regardless of the presence of the peptide. The quantity and location of visualized SPIO were directly related to the vascularity of the tumor, most of which appeared to reside at the tumor surface. This ability to visualize SPIO at the tumor is in part a function of tumor maturity (vascularity), where larger and more established tumors demonstrated SPIO accumulation at the tumor site in nearly all cases.

#### Persistence of Tumor SPIO-(SOR-C27)_x_ at 26 hours

The most interesting finding in the MRI aspect of this study was the differential persistence of the SOR-C27-SPIO compared to SPIO control injections. Both types of SPIO beads were present in tumors at 2 hours, whereas only the SOR-C27-SPIO beads could be seen at tumor sites by the next MRI time point at 26 hours (Figs. 5A-*iii* and 5B-*iii*). Based on Fig. S7, we note that for both fluorescence and MRI imaging studies, the time frame and dosage of the conjugated SOR-C27 is unlikely to have an observable effect upon the tumors.

#### Histological Confirmation of iron in tumor sections

The tumor from one SOR-C27-SPIO injection animal had sections stained with Prussian Blue for detection of the iron of the SPIO particles. Consistent with MRI observations of darkening within the tumor, Prussian Blue staining revealed the presence of SPIO particles. It was unclear whether the SPIO particles were within the tumor or perivascular to it.

## Discussion

TRPV6 channel expression is enhanced within numerous carcinomas, primary and metastatic (as reported within Refs [14] [19]–[23] and confirmed within a preliminarily investigation Fig. S8). Because silencing of TRPV6 using siRNA increased apoptosis and reduced proliferation, the channel has gained much attention as a therapeutic target [29]. Our results provide a proof-of-concept for the use of SOR peptides as a targeted delivery system to enhance tumor detection and therapy. Both SOR-C13 and SOR-C27 where shown to inhibit calcium influx in TRPV6 over-expressing cells and is more effective at reducing cell viability than cis-Platin as demonstrated from results of a previous investigation (Fig. S9). The peptides show high potency for a common TRPV6 binding site and, to our knowledge, are the first report of peptide inhibitors of this channel. Interestingly, the affinities of SOR-C13 and SOR-C27 for TRPV6 (IC_50_ = 14 and 65 nM, respectively) are approximately four orders of magnitude higher than that of recently reported small molecule inhibitors of TRPV6 [38]. The binding of SOR peptides to TRPV6 channels is use-dependent (*i.e*., TRPV6 must be in an open configuration). Thus, in whole-cell patch experiments, peptide binding to TRPV6 occurs when the channel is opened by the voltage ramp and during the ramp both SOR peptides exhibit slow washout rates, which could be advantageous for therapeutics and diagnostics. In addition, the route of metabolism and excretion of fluorescently tagged SOR-C27 involves the liver and kidney with greatly reduced processing in lymph nodes and at the tumor site. Unbound SOR peptides appear to be rapidly degraded and excreted, potentially minimizing the risk of off-target effects.

In addition to their binding to the TRPV6 channel, *per se*, NMR evidence suggests that SOR peptides are stabilized by membrane environments found in tumor cells. The SOR-C27 peptide does not exhibit a regular stable secondary structure in either buffered water or zwitterionic DPC micelle solution (a model for mammalian cell membranes). Instead, SOR-C27 bound strongly to anionic SDS micelles (a model for cancer and bacterial cell membranes) and formed a short helical structure, a property that could favor preferential binding of this peptide to carcinoma cell membranes.

The major translational significance of this investigation is the demonstrated bioaccumulation and tumor homing ability of the SOR peptides to human ovarian tumor sites in xenograft mouse models using fluorescent imaging and MRI approaches. Conjugation of SOR-C27 *via* its cysteine residue with Cy5.5 reduced the binding affinity for TRPV6 measured in whole-cell patch experiments. Although not measured, it would seem likely that conjugation of SOR-C27 to SPIO particles would also result in some loss of peptide affinity for TRPV6. Nonetheless, we demonstrated that tagged SOR-C27 peptides had an extended persistence at the tumor site over the control (at least 24 hours for the SOR-C27 with fluorescent and MRI investigations; possibly 72 hours for the SOR-C13 with fluorescent studies, preliminary data not shown), and that the peptide exhibited a capacity to selectively bind *in vivo* to human ovarian and prostate tumors. The ability of the SOR-C27 to bind tumors *in vivo* reflects the biologically relevant high levels of TRPV6 channels within the xenografts as demonstrated by excised xenograft tumors derived from SKOV-3 cells (Fig. S8).

With detection concentrations in the µM range, SPIO is one of the most sensitive MR contrast agents and is appealing for diagnostic probe development. Unfortunately, SPIO is a negative contrast agent producing dark voids in MRI images near SPIO deposits. These dark voids can be confounded by background voids from image artifacts produced by local main magnetic field distortions. Although not employed in this study, a number of techniques have recently been developed for detection of SPIO using positive contrast MR pulse sequences [39], [40]. More generally, these would eliminate background signal voids and be sensitive to SPIO only, thus alleviating difficulties associated with detection of metastatic disease. Regardless, for this study, the pre-contrast injection baseline scans were used to differentiate background signal voids from those induced by SPIO. Additionally, our primary finding of SPIO detection in tumors show signal voids at 2 hours post-contrast injection that are not present in the pre-contrast baselines, and which are eliminated at 26 hours post-injection in control SPIO scans. The detection of SPIO voids in lymph nodes at 26 hours in all scans is a positive control for SPIO detection in all cases.

Conjugated SOR peptides may be of utility for detecting (*via* MRI contrast agent) tumors and delivering diagnostic or therapeutic payloads (*e.g.* chemotherapeutic substances) specifically and directly to the TRPV6-rich tumor of ovarian and potentially others such as breast, thyroid, prostate and colon as well as certain leukemia's and lymphomas (Fig. S9). Interestingly from our previous investigations of cell viability and tumor growth, the non-conjugated peptides maybe of therapeutic interest. The degree of cell viability (*i.e.* growth inhibition) for SOR-C13 and SOR-C27 on 12 cancer cell lines is consistently reduced in some cases comparable to cis-Platin (Fig. S9). It should be noted that the time frame of the study is 72 hours consistent with a physiological effect (*i.e.* depletion of calcium concentration inducing apoptosis) rather than a cytotoxic effect. In addition, SOR-C27 reduced the size of SKOV-3 xenograft tumors *in vivo* comparable to the current aggressive therapeutics for ovarian cancer (Fig. S7). This could open the door to a targeted approach for the early detection and treatment of ovarian cancer, where currently there is no reliable non-invasive diagnostic detection or therapeutic. The studies reported here have led to the entry of the unconjugated SOR-C13 peptide into a Phase I human clinical trial looking at its tolerability and safety in patients with advanced cancers over-expressing TRPV6.

## Supporting Information

Figure S1 **SOR-C13-mediated reduction of TRPV6 current amplitude in TRPV6/EGFP HEK-293 cells.**
(PDF)

Figure S2 **Volumetric images of mice bearing ovarian and prostate xenografts.**
(PDF)

Figure S3 ***Ex vivo*** **optical images of various organs from mice with SKOV-3 xenograft tumors.**
(PDF)

Figure S4 **Representative axial MRI image of mouse bearing a SKOV-3 xenograft tumor.**
(PDF)

Figure S5 **Representative axial and saggital MRI images of xenograft mouse with SPIO control.**
(PDF)

Figure S6 **Magnified representative axial, saggital and transverse MRI images of a xenograft mouse with SOR-C27-SPIO injection.**
(PDF)

Figure S7 **Comparison of the growth curves of SKOV-3 derived tumors in NOD/SCID mice treated with SOR-C27 or CAT.**
(PDF)

Figure S8 **Western Blot band density comparison of lysates from xenografted primary human ovarian tumors and a commercial lysate of healthy ovary.**
(PDF)

Figure S9 **Inhibition of cancer cell line growth by SOR-C13, SOR-C27 and cis-Platin after 72 hr of exposure.**
(PDF)

## References

[1] Rossing MA, Wicklund KG, Cushing-Haugen KL, Weiss NS (2010) Predictive value of symptoms for early detection of ovarian cancer. J Natl Cancer Inst 102: 222–229. 20110551 10.1093/jnci/djp500 PMC2826180

[2] Howlader N, Noone AM, Krapcho M, Neyman N, Aminou R, et al.. (2012) SEER Cancer Statistics Review, 1975–2009 (Vintage 2009 Populations). National Cancer Institute. Bethesda, MD.

[3] Hoenderop JG, van der Kemp AW, Hartog A, van de Graaf SF, van Os CH, et al (1999) Molecular identification of the apical Ca^2+^ channel in 1, 25-dihydroxyvitamin D3-responsive epithelia. J Biol Chem 274: 8375–8378. 10085067 10.1074/jbc.274.13.8375

[4] Peng JB, Chen XZ, Berger UV, Vassilev PM, Tsukaguchi H, et al (1999) Molecular cloning and characterization of a channel-like transporter mediating intestinal calcium absorption. J Biol Chem 274: 22739–22746. 10428857 10.1074/jbc.274.32.22739

[5] Caterina MJ, Schumacher MA, Tominaga M, Rosen TA, Levine JD, et al (1997) The capsaicin receptor: a heat-activated ion channel in the pain pathway. Nature 389: 816–824. 9349813 10.1038/39807

[6] Montell C (2003) The venerable inveterate invertebrate TRP channels. Cell Calcium 33: 409–417. 12765686 10.1016/s0143-4160(03)00053-8

[7] Montell C (2011) The history of TRP channels, a commentary and reflection. Pflugers Arch 461: 499–506. 21287198 10.1007/s00424-010-0920-3

[8] Montell C, Rubin GM (1989) Molecular characterization of the Drosophila trp locus: a putative integral membrane protein required for phototransduction. Neuron 2: 1313–1323. 2516726 10.1016/0896-6273(89)90069-x

[9] Gees M, Colsoul B, Nilius B (2010) The role of transient receptor potential cation channels in Ca^2+^ signaling. Cold Spring Harb Perspect Biol 2: a003962. 20861159 10.1101/cshperspect.a003962 PMC2944357

[10] Pedersen SF, Owsianik G, Nilius B (2005) TRP channels: an overview. Cell Calcium 38: 233–252. 16098585 10.1016/j.ceca.2005.06.028

[11] Wu LJ, Sweet TB, Clapham DE (2010) International Union of Basic and Clinical Pharmacology. LXXVI. Current progress in the mammalian TRP ion channel family. Pharmacol Rev 62: 381–404. 20716668 10.1124/pr.110.002725 PMC2964900

[12] Nijenhuis T, Hoenderop JG, Nilius B, Bindels RJ (2003) (Patho)physiological implications of the novel epithelial Ca^2+^ channels TRPV5 and TRPV6. Pflugers Arch 446: 401–409. 12748856 10.1007/s00424-003-1038-7

[13] Nijenhuis T, Hoenderop JG, van der Kemp AW, Bindels RJ (2003) Localization and regulation of the epithelial Ca^2+^ channel TRPV6 in the kidney. J Am Soc Nephrol 14: 2731–2740. 14569082 10.1097/01.asn.0000094081.78893.e8

[14] Zhuang L, Peng JB, Tou L, Takanaga H, Adam RM, et al (2002) Calcium-selective ion channel, CaT1, is apically localized in gastrointestinal tract epithelia and is aberrantly expressed in human malignancies. Lab Invest 82: 1755–1764. 12480925 10.1097/01.lab.0000043910.41414.e7

[15] Bödding M (2007) TRP proteins and cancer. Cell Signal 19: 617–624. 17029734 10.1016/j.cellsig.2006.08.012

[16] Nilius B, Owsianik G, Voets T, Peters JA (2007) Transient receptor potential cation channels in disease. Physiol Rev 87: 165–217. 17237345 10.1152/physrev.00021.2006

[17] Prevarskaya N, Zhang L, Barritt G (2007) TRP channels in cancer. Biochim Biophys Acta 1772: 937–946. 17616360 10.1016/j.bbadis.2007.05.006

[18] Santoni G, Farfariello V, Amantini C (2011) TRPV channels in tumor growth and progression. Adv Exp Med Biol 704: 947–967. 21290335 10.1007/978-94-007-0265-3_49

[19] Bödding M, Wissenbach U, Flockerzi V (2002) The Recombinant Human TRPV6 Channel Functions as Ca^2+^Sensor in Human Embryonic Kidney and Rat Basophilic Leukemia Cells. J Biol Chem 277: 36656–36664. 12138163 10.1074/jbc.M202822200

[20] Peng JB, Chen XZ, Berger UV, Weremowicz S, Morton CC, et al (2000) Human calcium transport protein CaT1. Biochem Biophys Res Commun 278: 326–332. 11097838 10.1006/bbrc.2000.3716

[21] Peng JB, Zhuang L, Berger UV, Adam RM, Williams BJ, et al (2001) CaT1 expression correlates with tumor grade in prostate cancer. Biochem Biophys Res Commun 282: 729–734. 11401523 10.1006/bbrc.2001.4638

[22] Semenova SB, Vassilieva IO, Fomina AF, Runov AL, Negulyaev YA (2009) Endogenous expression of TRPV5 and TRPV6 calcium channels in human leukemia K562 cells. Am J Physiol Cell Physiol 296: C1098–1104. 19295174 10.1152/ajpcell.00435.2008

[23] Wissenbach U, Niemeyer BA, Fixemer T, Schneidewind A, Trost C, et al (2001) Expression of CaT-like, a novel calcium-selective channel, correlates with the malignancy of prostate cancer. J Biol Chem 276: 19461–19468. 11278579 10.1074/jbc.M009895200

[24] Fixemer T, Wissenbach U, Flockerzi V, Bonkhoff H (2003) Expression of the Ca^2+^-selective cation channel TRPV6 in human prostate cancer: a novel prognostic marker for tumor progression. Oncogene 22: 7858–7861. 14586412 10.1038/sj.onc.1206895

[25] Wissenbach U, Niemeyer B, Himmerkus N, Fixemer T, Bonkhoff H, et al (2004) TRPV6 and prostate cancer: cancer growth beyond the prostate correlates with increased TRPV6 Ca^2+^ channel expression. Biochem Biophys Res Commun 322: 1359–1363. 15336984 10.1016/j.bbrc.2004.08.042

[26] Schwarz EC, Wissenbach U, Niemeyer BA, Strauss B, Philipp SE, et al (2006) TRPV6 potentiates calcium-dependent cell proliferation. Cell Calcium 39: 163–173. 16356545 10.1016/j.ceca.2005.10.006

[27] Lehen'kyi V, Flourakis M, Skryma R, Prevarskaya N (2007) TRPV6 channel controls prostate cancer cell proliferation via Ca^2+^/NFAT-dependent pathways. Oncogene 26: 7380–7385. 17533368 10.1038/sj.onc.1210545

[28] Bolanz KA, Hediger MA, Landowski CP (2008) The role of TRPV6 in breast carcinogenesis. Mol Cancer Ther 7: 271–279. 18245667 10.1158/1535-7163.MCT-07-0478

[29] Peters AA, Simpson PT, Bassett JJ, Lee JM, Da Silva L, et al (2012) Calcium channel TRPV6 as a potential therapeutic target in estrogen receptor-negative breast cancer. Mol Cancer Ther 11: 2158–2168. 22807578 10.1158/1535-7163.MCT-11-0965

[30] Stewart JM, Steeves BJ, Vernes K (2006) Paralytic peptide for use in neuromuscular therapy. USPTO. Pat no. 7,119,168.

[31] Stewart JM (2011) Peptide composition for cancer treatment by inhibiting TRPV6 calcium channel activity. USPTO. Appl No 12/886397.

[32] Kim DG, Kang HM, Jang SK, Shin HS (1992) Construction of a bifunctional mRNA in the mouse by using the internal ribosomal entry site of the encephalomyocarditis virus. Mol Cell Biol 12: 3636–3643. 1321342 10.1128/mcb.12.8.3636-3643.1992 PMC364630

[33] Al-Ansary D, Bogeski I, Disteldorf BM, Becherer U, Niemeyer BA (2010) ATP modulates Ca^2+^ uptake by TRPV6 and is counteracted by isoform-specific phosphorylation. FASEB J 24: 425–435. 19805577 10.1096/fj.09-141481

[34] Bödding M, Flockerzi V (2004) Ca^2+^ dependence of the Ca^2+^-selective TRPV6 channel. J Biol Chem 279: 36546–36552. 15184369 10.1074/jbc.M404679200

[35] Kovacs G, Montalbetti N, Simonin A, Danko T, Balazs B, et al (2012) Inhibition of the human epithelial calcium channel TRPV6 by 2-aminoethoxydiphenyl borate (2-APB). Cell Calcium 52: 468–480. 23040501 10.1016/j.ceca.2012.08.005

[36] Clinchy B, Gazdar A, Rabinovsky R, Yefenof E, Gordon B, et al (2000) The growth and metastasis of human, HER-2/neu-overexpressing tumor cell lines in male SCID mice. Breast Cancer Res Treat 61: 217–228. 10965998 10.1023/a:1006494001861

[37] Zheng D, Li D, Lu X, Feng Z (2010) Enhanced antitumor efficiency of docetaxel-loaded nanoparticles in a human ovarian xenograft model with lower systemic toxicities by intratumoral delivery. Oncol Rep 23: 717–724. 20127011 10.3892/or_00000689

[38] Landowski CP, Bolanz KA, Suzuki Y, Hediger MA (2011) Chemical inhibitors of the calcium entry channel TRPV6. Pharm Res 28: 322–330. 21057859 10.1007/s11095-010-0249-9

[39] Mani V, Briley-Saebo KC, Itskovich VV, Samber DD, Fayad ZA (2006) Gradient echo acquisition for superparamagnetic particles with positive contrast (GRASP): sequence characterization in membrane and glass superparamagnetic iron oxide phantoms at 1.5T and 3T. Magn Reson Med 55: 126–135. 16342148 10.1002/mrm.20739

[40] Stuber M, Gilson WD, Schar M, Kedziorek DA, Hofmann LV, et al (2007) Positive contrast visualization of iron oxide-labeled stem cells using inversion-recovery with ON-resonant water suppression (IRON). Magn Reson Med 58: 1072–1077. 17969120 10.1002/mrm.21399

