# Supplementary material for: In Vivo Detection of Human TRPV6-Rich Tumors with Anti-Cancer Peptides Derived from Soricidin
Source: PLoS One. 2013 Mar 15;8(3):e58866. doi: 10.1371/journal.pone.0058866 (PMC3598914; doi:10.1371/journal.pone.0058866)
Supplement: Figure S1 — SOR-C13-mediated reduction of TRPV6 current amplitude in TRPV6/EGFP HEK-293 cells. (PDF) [file pone.0058866.s001.pdf]

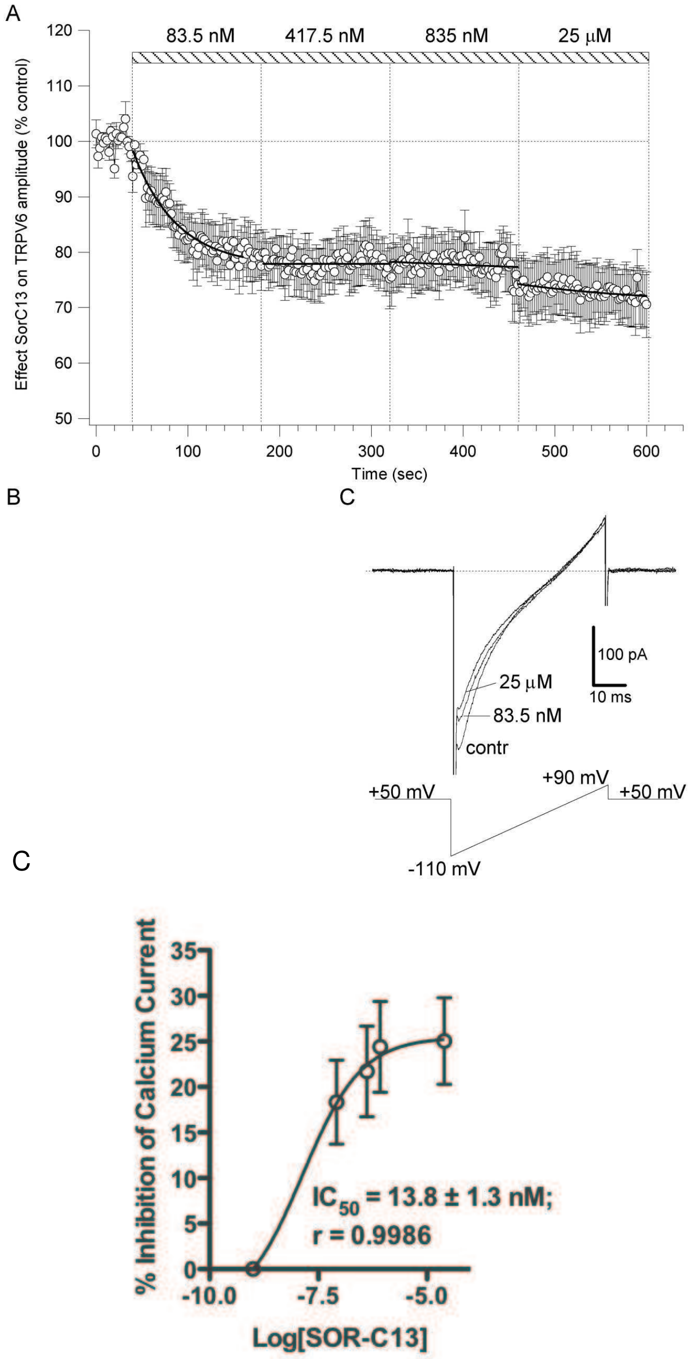

Figure S1. SOR-C13-mediated reduction of TRPV6 current amplitude in TRPV6/EGFP HEK-293 cells. (A) Time course of the SOR-C13-induced reduction in TRPV6 current amplitude. Normalized TRPV6 current amplitudes (%) are plotted as a function of time for SOR-C13 concentrations of 83.5 nM (n = 9), 417.5 nM (n = 9), 835 nM (n = 7) and 25 μM (n = 7). The gray bar indicates the period of SOR-C13 application. **(B)** Examples of traces of TRPV6 currents. Each trace is an average of 10 traces. **(C)** Dose-response curve for SOR-C13. Error bars represent SEM.
