# Supplementary material for: In Vivo Detection of Human TRPV6-Rich Tumors with Anti-Cancer Peptides Derived from Soricidin
Source: PLoS One. 2013 Mar 15;8(3):e58866. doi: 10.1371/journal.pone.0058866 (PMC3598914; doi:10.1371/journal.pone.0058866)
Supplement: Figure S2 — Volumetric images of mice bearing ovarian and prostate xenografts. (PDF) [file pone.0058866.s002.pdf]

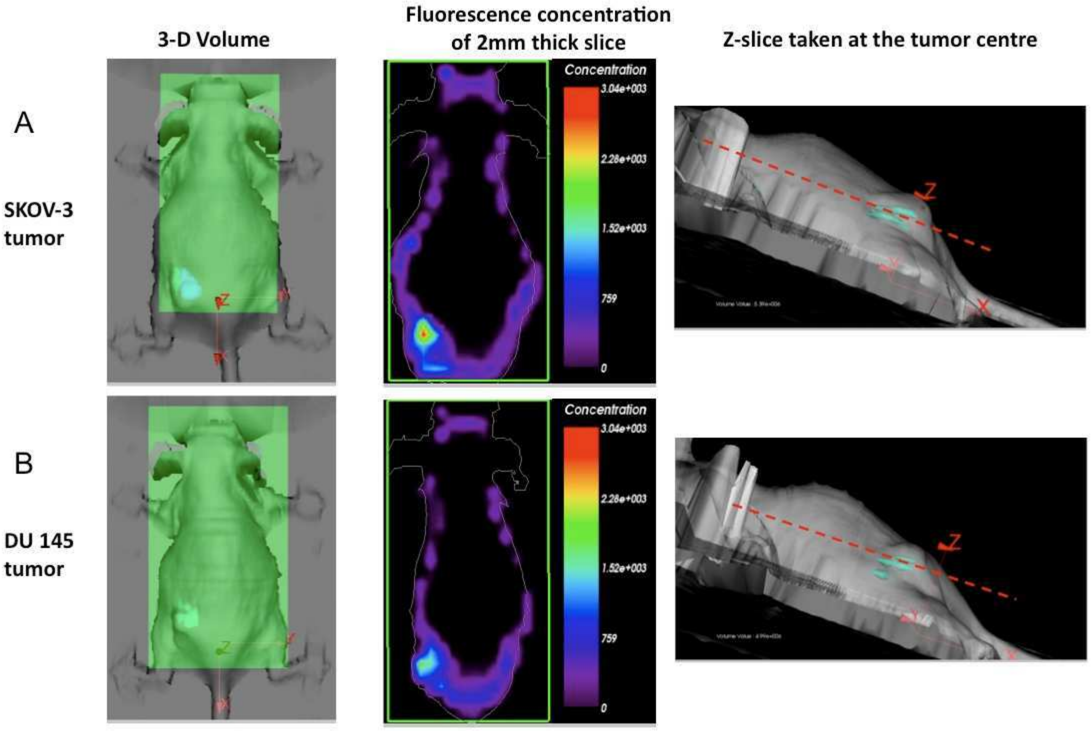


**Figure S2. Volumetric images of mice bearing ovarian and prostate xenografts.** Volumetric images of mice bearing **(A)** ovarian and **(B)** prostate xenografts were taken at about 3 hours post-injection of SOR-C27-Cy5.5. A 2 mm digital Z-slice was obtained at 3 hours to match the maximal tumor fluorescence. This technique allows discrimination of the tumor fluorescence from the fluorescence observed in kidneys.
