# Supplementary material for: In Vivo Detection of Human TRPV6-Rich Tumors with Anti-Cancer Peptides Derived from Soricidin
Source: PLoS One. 2013 Mar 15;8(3):e58866. doi: 10.1371/journal.pone.0058866 (PMC3598914; doi:10.1371/journal.pone.0058866)
Supplement: Figure S3 — Ex vivo optical images of various organs from mice with SKOV-3 xenograft tumors. (PDF) [file pone.0058866.s003.pdf]

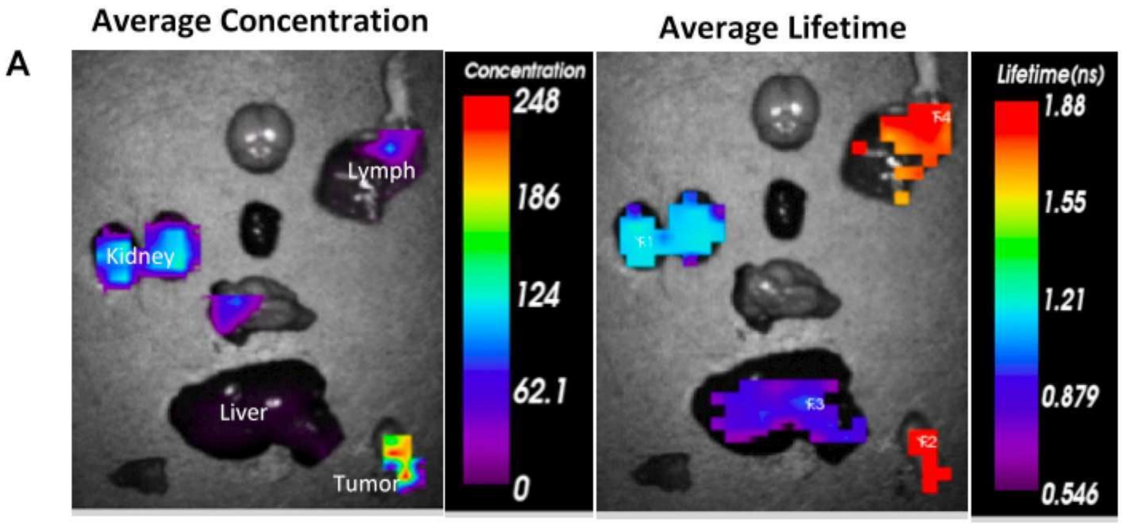

Figure S3. ***Ex vivo*** **optical images of various organs from mice with SKOV-3 xenograft tumors.** *Ex vivo* optical images representing the average fluorescence concentration of SOR-C27-Cy5.5 (left panel) and the average lifetime of the fluorescence signal (right panel) in various organs from mice with SKOV-3 xenograft tumors 24 hours after injection.
