# Supplementary material for: In Vivo Detection of Human TRPV6-Rich Tumors with Anti-Cancer Peptides Derived from Soricidin
Source: PLoS One. 2013 Mar 15;8(3):e58866. doi: 10.1371/journal.pone.0058866 (PMC3598914; doi:10.1371/journal.pone.0058866)
Supplement: Figure S4 — Representative axial MRI image of mouse bearing a SKOV-3 xenograft tumor. (PDF) [file pone.0058866.s004.pdf]

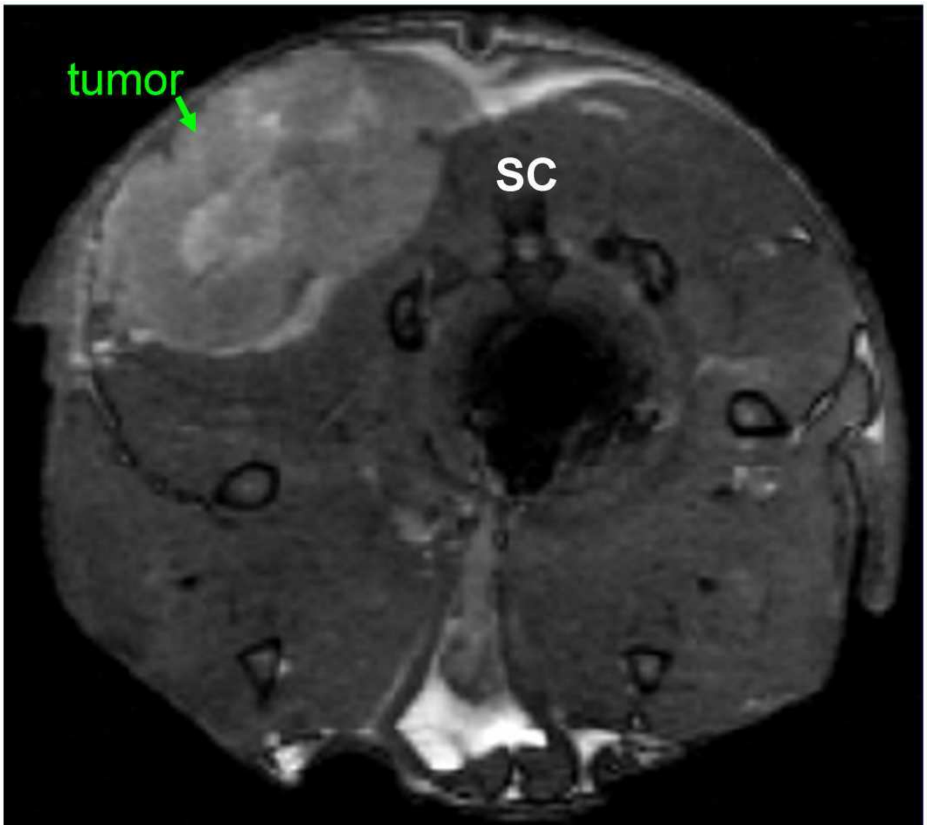

Figure S4. Representative axial MRI image of mouse bearing a SKOV-3 xenograft tumor. $(150\ \mu m)^3$ axial MRI image showing a representative mouse bearing a SKOV-3 xenograft tumor scanned 58 days post cell implantation; $V_{tumor} = 296.0\ mm^3$ (SC=spinal cord).
