# Supplementary material for: In Vivo Detection of Human TRPV6-Rich Tumors with Anti-Cancer Peptides Derived from Soricidin
Source: PLoS One. 2013 Mar 15;8(3):e58866. doi: 10.1371/journal.pone.0058866 (PMC3598914; doi:10.1371/journal.pone.0058866)
Supplement: Figure S5 — Representative axial and saggital MRI images of xenograft mouse with SPIO control. (PDF) [file pone.0058866.s005.pdf]

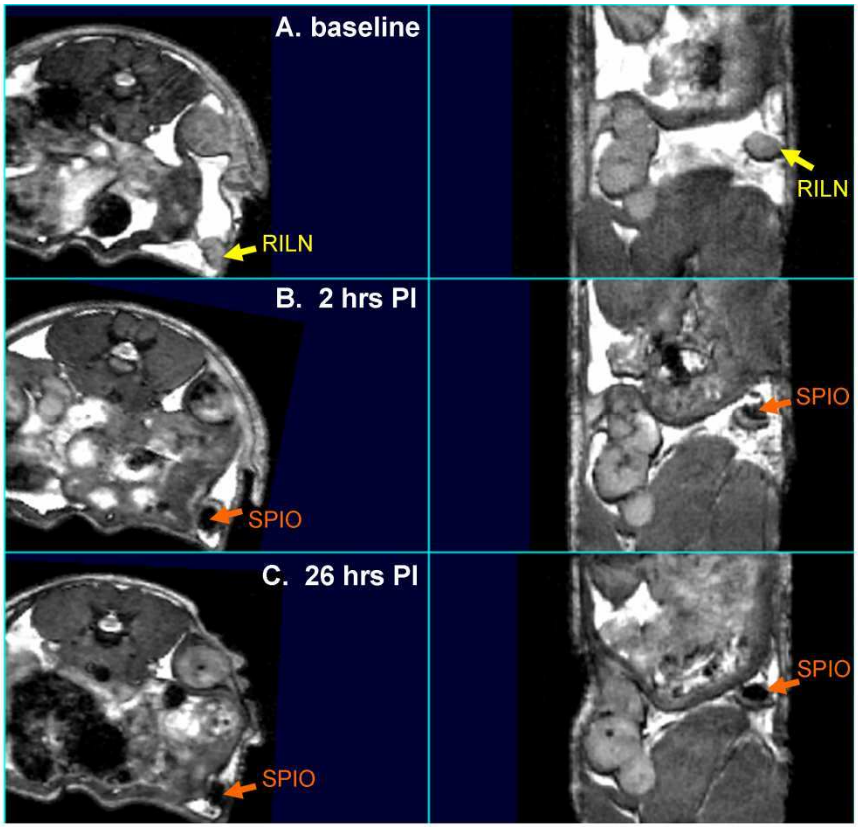

Figure S5. Representative axial and saggital MRI images of xenograft mouse with SPIO control. $(150\ \mu m)^3$ axial and saggital MRI images of mouse with control SPIO injection. (**A**) right inguinal lymph node (yellow arrow) at the baseline scan, (**B**) the presence of SPIO is seen at 2 hours as indicated by dark signal void in the inguinal lymph node, and (**C**) SPIO still remains at 26 hours post injection. The same trend was observed on the contralateral lymph node.
