# Supplementary material for: In Vivo Detection of Human TRPV6-Rich Tumors with Anti-Cancer Peptides Derived from Soricidin
Source: PLoS One. 2013 Mar 15;8(3):e58866. doi: 10.1371/journal.pone.0058866 (PMC3598914; doi:10.1371/journal.pone.0058866)
Supplement: Figure S6 — Magnified representative axial, saggital and transverse MRI images of a xenograft mouse with SOR-C27-SPIO injection. (PDF) [file pone.0058866.s006.pdf]

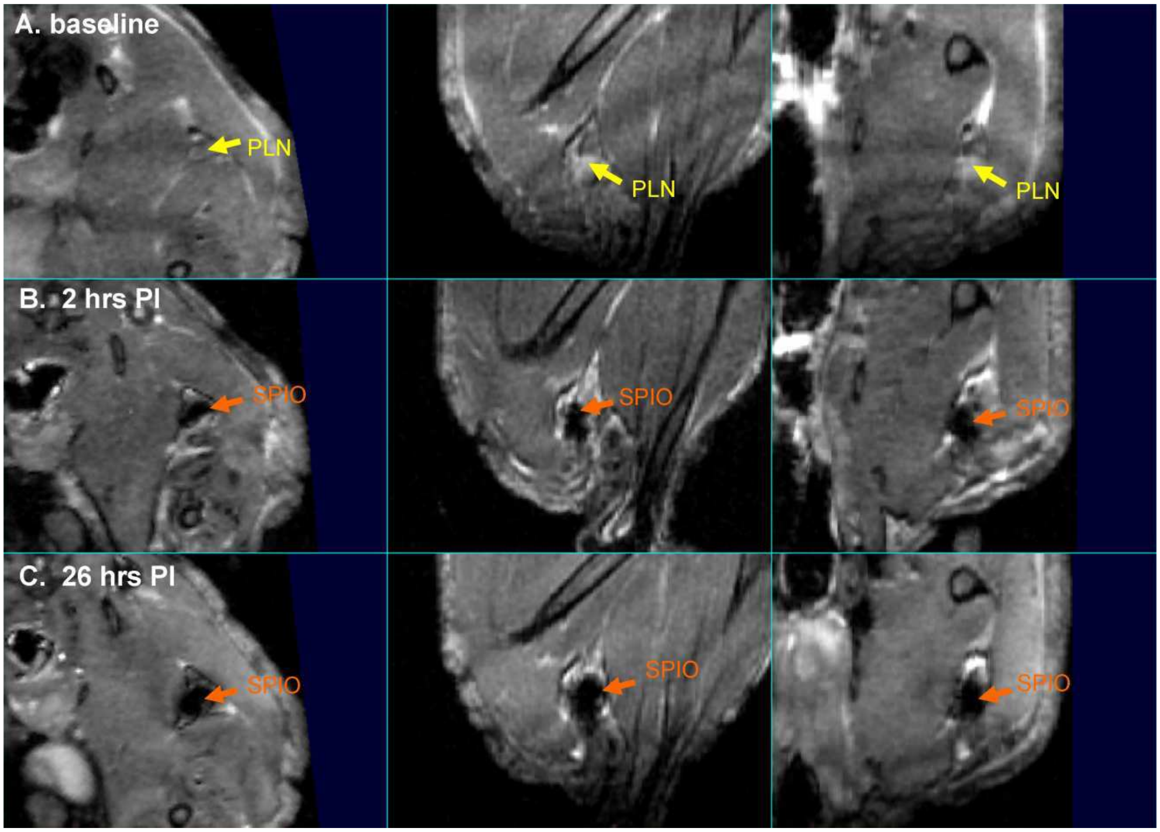

Figure S6. Magnified representative axial, saggital and transverse MRI images of a xenograft mouse with SOR-C27-SPIO injection. (150 μm)^3 (magnified) axial, saggital and transverse MRI images of a mouse with SOR-C27-SPIO injection showing (**A**) right popliteal lymph node (yellow arrow) at baseline scan, (**B**) SPIO is seen at 2 hours as indicated by dark signal void in the popliteal lymph node, and (**C**) SOR-C27-SPIO still remains at 26 hours post injection. Again the same trend was observed on the contralateral lymph node.
