# Supplementary material for: In Vivo Detection of Human TRPV6-Rich Tumors with Anti-Cancer Peptides Derived from Soricidin
Source: PLoS One. 2013 Mar 15;8(3):e58866. doi: 10.1371/journal.pone.0058866 (PMC3598914; doi:10.1371/journal.pone.0058866)
Supplement: Figure S7 — Comparison of the growth curves of SKOV-3 derived tumors in NOD/SCID mice treated with SOR-C27 or CAT. (PDF) [file pone.0058866.s007.pdf]

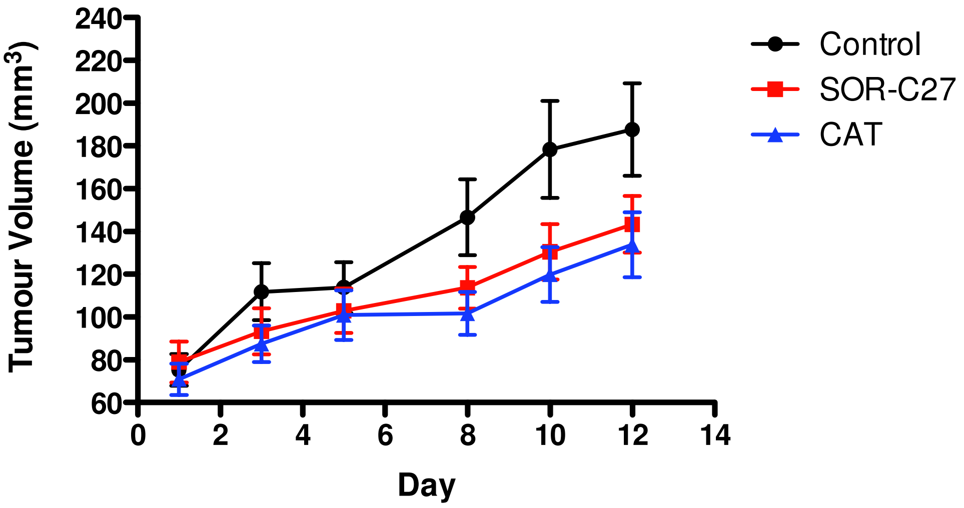

Figure S7: Comparison of the growth curves of SKOV-3 derived tumors in NOD/SCID mice treated with SOR-C27 or CAT. A comparison of the growth curves of SKOV-3 derived tumors in NOD/SCID mice treated daily with SOR-C27 (400 mg/kg) or the prescribed weekly dose of the aggressive CAT (carboplatin, 50 mg/kg and paclitaxel, 18 mg/kg) with controls receiving no treatment. The values are the mean ± SEM, $n = 20 – 24$. Six weeks after SKOV-3 cell injection, tumor sizes were measured and mice were randomly grouped into 5 or 6 mice per group with 2-4 grafts per mouse. SOR-C27 was administered at 400 mg/kg i.p. daily for 12 days; mice in the control group received saline. A combination of carboplatin (50 mg/kg) and paclitaxel (18 mg/kg) was used as a positive control. This dose of CAT is in the lower range of the therapeutic response range and was given once per week by i.p. injection. Two-way ANOVA indicated a significant difference in the growth curves between the control and the two treatments ($p < 0.0001$) but no statistical difference between SOR-C27 and CAT treatment ($p = 0.2178$). Information derived from patent US 8,211,857.
