# Supplementary material for: In Vivo Detection of Human TRPV6-Rich Tumors with Anti-Cancer Peptides Derived from Soricidin
Source: PLoS One. 2013 Mar 15;8(3):e58866. doi: 10.1371/journal.pone.0058866 (PMC3598914; doi:10.1371/journal.pone.0058866)
Supplement: Figure S8 — Western Blot band density comparison of lysates from xenografted primary human ovarian tumors and a commercial lysate of healthy ovary. (PDF) [file pone.0058866.s008.pdf]

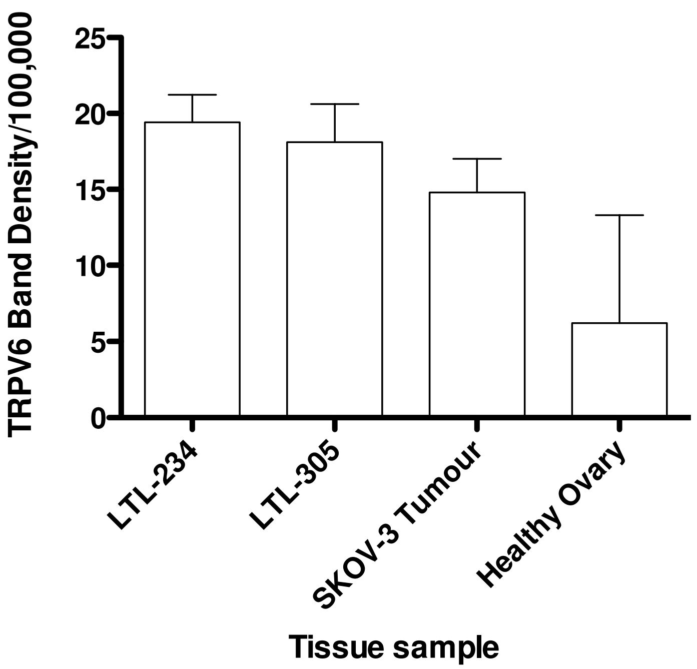

Figure S8: Western Blot band density comparison of lysates from xenografted primary human ovarian tumors and a commercial lysate of healthy ovary. A comparison of Western Blot band densities from lysates of xenografted primary human ovarian tumors (a mucinous carcinoma, LTL-234 and a clear cell carcinoma, LTL-305; Living Tumor Laboratory, British Columbia Cancer Research Centre), SKOV-3 derived xenografts, and a commercial lysate of healthy ovary (Clontech, cat#635308, lot#1006234A). The values are mean ± SEM, n = 11 – 15. Tumors LTL-234 and LTL-305 showed 3.1 and 2.9 greater band density in Western Blots than healthy human ovary tissue. SKOV-3 derived control tumors showed 2.4 fold greater band density than healthy tissue. The healthy sample varied considerably (~20%) and typically showed very low band density. These data serve to indicate that TRPV6 is commonly expressed in ovarian cancers.
