# Supplementary material for: In Vivo Detection of Human TRPV6-Rich Tumors with Anti-Cancer Peptides Derived from Soricidin
Source: PLoS One. 2013 Mar 15;8(3):e58866. doi: 10.1371/journal.pone.0058866 (PMC3598914; doi:10.1371/journal.pone.0058866)
Supplement: Figure S9 — Inhibition of cancer cell line growth by SOR-C13, SOR-C27 and cis-Platin after 72 hr of exposure. (PDF) [file pone.0058866.s009.pdf]

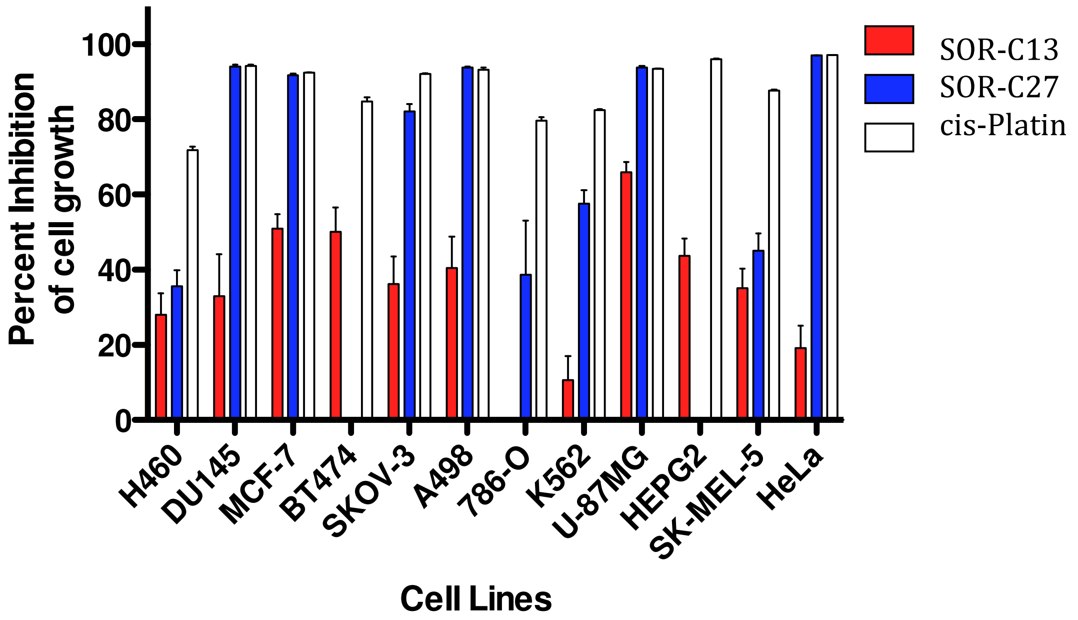

Figure S9: Inhibition of cancer cell line growth by SOR-C13, SOR-C27 and cis-Platin after 72 hr of exposure. The concentration of peptides was 100 uM (except where noted) and cis-Platin 250 uM. The following were situations were not at 100 uM peptide: MCF-7, SOR-C13 at 10 uM; SK-MEL-5 SOR-C13 at 50 uM; U-87MG SOR-C27 at 50 uM; HeLa, SOR-C27 at 50 uM. Data for SOR-C27 with BT474 and HEPG2 are not available. The value is the mean ± SEM, $n = 6 – 9$. All cell lines were obtained from ATCC and represented: non-small cell lung carcinoma (NCI-H460; ATCC #HTB-177), prostate carcinoma (DU145; ATCC #HTB-81), breast adenocarcinoma (MCF-7; ATCC #HTB-22), breast ductal carcinoma (BT474; ATCC #HTB-20), ovarian adenocarcinoma (SK-OV-3, ATCC #HTB-77), kidney carcinoma (A-498, ATCC #HTB-44; 786-O, ATCC #CRL-1932), chronic myelogenous leukemia (K562; ATCC #CCL-243), brain glioblastoma (U-87MG, ATCC #HTB-14), hepatocellular carcinoma (HepG2, ATCC #HB-8605), skin melanoma (SK-MEL-5; ATCC #HTB-70) and cervix adenocarcinoma (HeLa; ATCC #CCL-2). The culture medium for each cell line was that recommended by ATCC. Control wells (6 for each cell line on three separate plates, total of 18) and exposure to test articles (3 wells for each cell line on three separate plates, total of 9) were measured for viability after 72 hr using Promega CellTiter Blue (Cat. No. G8081). Every 24 hours, cell culture media were aspirated and fresh PBS or test article, dissolved in PBS, was added to each well of the plate. In the last 4 hours of the incubation, 20 ul of resazurin solution (Promega CellTiter-Blue kit) were added and the plates returned to CO_2 incubator. Plates were analyzed using a fluorescence microplate reader (Tecan Infinite M200 microplate reader) at 560 nm Ex/590 nm Em. Percentage of inhibition of cell proliferation (% growth inhibition) was calculated as the difference between the fluorescence value of vehicle-treated cells (Flveh) and fluorescence of test article-treated cells (FlTA). Information derived from patent US Patent #8,211,857 issued July 3, 2012.
